# Challenges with communication of critical laboratory results in a resource-limited setting in South Africa

**DOI:** 10.4102/ajlm.v13i1.2457

**Published:** 2024-09-23

**Authors:** Ameerah Davids, Annalise E. Zemlin, Elsie C. Kruger

**Affiliations:** 1 Department of Pathology, Division of Chemical Pathology, Faculty of Medicine and Health Sciences, Stellenbosch University, Cape Town, South Africa; 2 Department of Pathology, Division of Chemical Pathology, National Health Laboratory Service, Cape Town, South Africa

**Keywords:** laboratory critical results, patient outcome, patient safety, post-post analytical, communication

## Abstract

**Background:**

Critical laboratory results are test results suggesting a patient is in immediate danger unless treatment is administered promptly. There is a paucity of studies in sub-Saharan Africa on clinicians’ utility of these results and affected patients’ outcomes. In our resource-limited setting in South Africa, we rely on telephonic communication to convey critical results.

**Objective:**

The aim of this study was to determine the average time for clinicians to acknowledge these results on the laboratory information system and to determine the outcome of affected patients.

**Methods:**

A retrospective descriptive audit at Tygerberg Academic Hospital was conducted between 01 October 2021 and 31 March 2022. Critical results and the time of acknowledgement by clinicians on the laboratory information system were obtained from inpatients and outpatients. One hundred and twenty inpatient critical results were randomly selected for a folder review to determine patient outcome.

**Results:**

Overall, 2514 critical results were reported, and 63 results were excluded. The remaining 2451 results were obtained from 1346 patients. The majority (94.5%) of results were obtained from inpatients, where 1681 (68.6%) were acknowledged within 24 h. The folder audit of 120 patients determined that 40 (33.3%) patients demised. In 82 (68.3%) patients, communication of a critical result did not alter clinical management.

**Conclusion:**

Critical laboratory results are crucial to patient care. This study found that approximately one-third of critical laboratory results were not used within 24 h. Engaging clinicians in current practice and implementing a means of improved communication of critical results is required.

**What this study adds:**

The study adds to the evidence of challenges experienced with communicating critical results to clinicians which could impact patient care. This is especially true in resource-limited settings; clinicians need to be made aware of the importance of these results, and communication modes need to be improved.

## Introduction

Patient management is highly dependent on laboratory data, where already in 1996, Forsman claimed that approximately 70% of clinical decisions regarding diagnosis, treatment or prevention are based on laboratory results.^1^ Therefore, exploring the bridge between laboratory and clinical medicine and its impact on patient outcomes is crucial. A critical laboratory result was first described by Lundberg in 1972, and is defined as a laboratory test result that suggests a patient is in imminent danger unless appropriate intervention is given timeously.^2^ Critical result reporting is a parameter required for laboratory accreditation, where the International Organization for Standardization (ISO 15189:2022) states that a clinician or another authorised staff member must promptly be notified of critical results.^3^

The ‘brain-to-brain loop’, introduced by Lundberg in 1981, describes the nine processes involved in generating a laboratory result.^4,5^ These nine steps include test ordering, collection, identification, transportation, separation, analysis, reporting, interpretation, and action. The post-post analytical phase of laboratory testing consists of receiving the result, interpreting the test and, lastly, using the information for patient management by the clinician.^5,6^ Laboratory testing plays a vital role in patient care, and Lundberg later proposed a 10th step to the brain-to-brain loop – namely, patient outcome.^6^ Critical result reporting is one aspect of the post-analytical phase that may impact patient outcomes.

Globally, critical values are often institute specific and determined by expert committees that include laboratory staff and clinicians.^7,8^ In South Africa, the National Health Laboratory Service (NHLS) is the largest provider of clinical laboratory services and is the primary laboratory service used for public healthcare facilities.^9^ Within the NHLS, the chemical pathology expert committee determines the critical chemistry result values, which are nationally harmonised and regularly reviewed.

Patient safety is a crucial criterion used by regulatory bodies, and in 2009 the World Health Organization World Alliance for Patient Safety listed communication of critical test results as a potential solution.^10^ Despite the importance of these results regarding patient safety, issues related to their reporting and management are concerning. This includes the measures for evaluating and monitoring the outcomes of critical results which occur outside of the laboratory’s control.^11^

There is currently a paucity of data on the impact of critical laboratory results in the South African setting, where most public hospitals have limited information technology infrastructure. At Tygerberg Academic Hospital (TAH), clinicians complete physical request forms for laboratory testing and patient results are accessed via a web-based laboratory information system (LIS). Although critical results are not automatically communicated on the web system, a short message service (SMS) can be sent to clinicians’ cell phones to alert them of critical results. Unfortunately, this often does not occur due to certain limitations. Clinicians must register to access this system using their unique medical registration numbers which can in turn be used to track activity across the platform. Incomplete laboratory request forms lacking a registration number have no contact details assigned via LIS, preventing an SMS from being sent. Additionally, no registered contact number of the requesting clinician may be listed. Therefore, the TAH chemistry laboratory relies on telephonic communication with wards to notify clinicians of critical results, which is challenging. The aim of this study was to determine the average time for critical results to be acknowledged by clinicians on TrakCare^®^ and to review the outcome of patients with these results. Due to the critical nature of these results, rapid acknowledgement within approximately 4 h was expected.

## Methods

### Ethical considerations

This study was approved by the Stellenbosch University Ethics Review Board of the Faculty of Medicine and Health Sciences (reference number: S22/07/139) and was performed in accordance with the Declaration of Helsinki. A waiver of informed consent was obtained for retrospective data collection. Patient data were anonymised to maintain confidentiality and were stored on password-protected devices. Only the investigators of the study had access to the data.

### Study site

This retrospective audit was conducted at the Chemical Pathology Laboratory of the NHLS at TAH, Cape Town, South Africa. The NHLS laboratory delivers a 24-h service to TAH, public secondary hospitals and primary healthcare facilities and receives approximately 120 000 chemistry samples monthly. Tygerberg Academic Hospital comprises 1384 beds across various disciplines, including adult and paediatric intensive care, high-care and general care units, and offers outpatient services. Additionally, it serves as the teaching hospital for the Faculty of Medicine and Health Sciences, Stellenbosch University. Critical results are communicated telephonically to both inpatient and outpatient departments from the NHLS laboratory by qualified pathologists, registrars, and medical technologists.

### Data collection

All chemistry critical results for inpatients and outpatients at TAH between 01 October 2021 and 31 March 2022 were obtained from the NHLS central data warehouse. The data were extracted from the LIS (InterSystems TrakCare^®^ Lab Enterprise, Cambridge, Massachusetts, United States) into Microsoft Office Excel (Microsoft Corporation, Redmond, Washington, United States). The data obtained included patient age, sex, requesting ward, critical laboratory result, critical result reporting time (time the critical result was communicated), time of clinician acknowledgement, and the requesting clinician’s registration status on TrakCare^®^. The results with an automatic SMS sent were obtained from the LIS, which automatically records whether this was successfully sent to the clinician, as well as time to acknowledgement obtained as described above. In addition, turn-around times were calculated and captured. Turn-around time in the laboratory is defined as the time from sample registration to result verification, in line with standard practice. Results viewed within 24 h were used for statistical analysis as we expected outpatient results to have a prolonged time, being communicated during business hours only (8:00 – 16:00). Thus, results viewed after 24 h were excluded from statistical analyses. Tygerberg Academic Hospital chemistry laboratory has no quality indicators for the time taken for clinicians to acknowledge critical results on TrakCare^®^.

### Folder review for patient outcome

The critical inpatient results were randomised and 120 of these were selected for a folder review. Clinical information was obtained from patient records, which were accessed using TAH’s Enterprise Content Manager system. The records were used to extract data regarding patient diagnosis, the use of point-of-care testing devices, whether patient management was changed once critical results were acknowledged, and patient outcomes. Critical results acknowledged after 24 h were excluded from the folder review.

### Data analysis

After extraction, the data were analysed using Excel^®^ and Statistical Package for Social Sciences version 27 (IBM Corp., Armonk, New York, United States), in consultation with the Biostatistics Division at the University of Stellenbosch. A Mann-Whitney *U* test was performed to evaluate whether there was a difference in the time taken to acknowledge a critical result between telephonic communication compared to SMS. A *p*-value < 0.05 was considered statistically significant. Critical results acknowledged on the TrakCare^®^ within 24 h of being communicated were used to calculate the median time to view results (in minutes).

## Results

### All critical results

**FIGURE 1:**
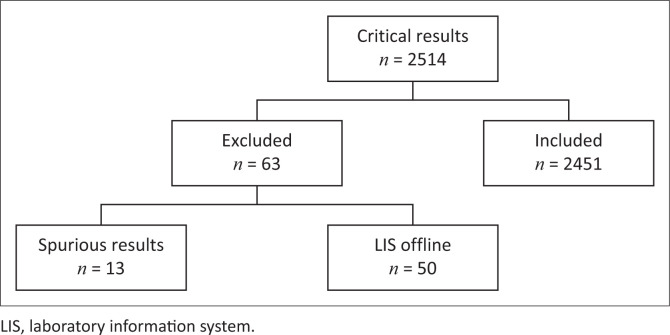
Critical results obtained over 6 months at Tygerberg Academic Hospital, South Africa, October 2021 to March 2022.

During the study period there were 2514 critical results. Sixty-three results were excluded from the analysis; 13 results were spurious, and 50 results were registered while the laboratory system was offline, and clinicians were unable to access the LIS to view results. The remaining 2451 critical results were obtained from 1346 patients (Figure 1).

The sex was equally distributed amongst the study population: 680 female patients (50.5%) and 664 male patients (49.3%). Table 1 depicts the age distribution of the patients, where 69.7% of results were obtained from patients aged 18 years and older.

**TABLE 1:** Demographic of patients with critical results at Tygerberg Academic Hospital, South Africa, October 2021 to March 2022.

Variable	Total (*N* = 1346)		Chart review (*N* = 120)	
	*n*	%	*n*	%
**Sex**				
Female	680	50.5	59	49.2
Male	664	49.3	61	50.8
Unknown	2	0.2	-	-
**Age (years)**				
0–1	316	23.5	33	27.5
2–17	92	6.8	7	5.8
18–50	514	38.2	47	39.2
> 50	424	31.5	33	27.5

**FIGURE 2:**
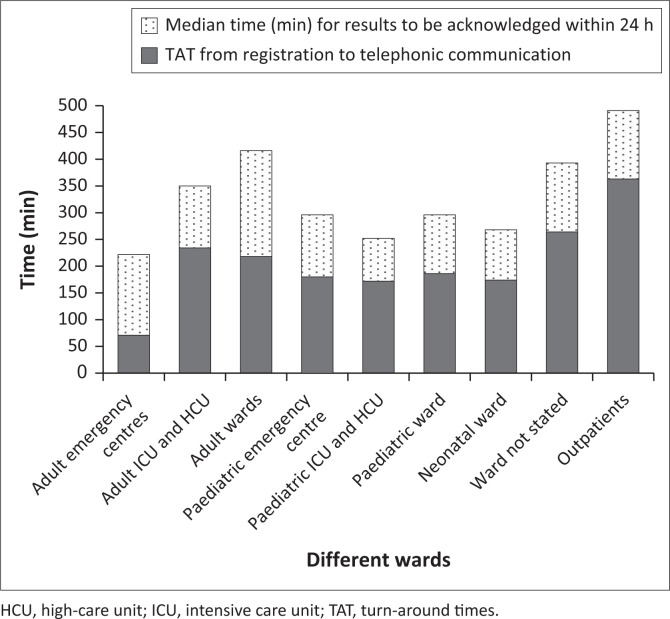
Time distribution from requesting wards at Tygerberg Academic Hospital, South Africa, October 2021 to March 2022.

Most critical results (*n* = 2316, 94.5%) were from inpatients. In 69 (2.8%) samples, the patient’s location was not recorded on the laboratory request form. These results had the longest inpatient laboratory turn-around time (262 min) but did not have the longest time to clinician confirmation compared to the other wards. Of the 2451 included critical results, 68.6% (1681/2451) were viewed within 24 h; the median time to acknowledgement ranged from 80 to 198 min across the different wards. The paediatric intensive care unit and high-care unit had the lowest median time for reviewing critical results (Table 2). The time taken from samples being registered in our laboratory to the result being communicated was up to 4 h in certain inpatient wards (Figure 2).

**TABLE 2:** Critical results from requesting wards at Tygerberg Academic Hospital, South Africa, October 2021 to March 2022.

Ward	Total results (*N* = 2451)		Number of patients (*N* = 1346)	
	*n*	%	*n*	%
**Adult**				
Emergency centre	422	17.2	310	23.0
ICU and HCU	314	12.8	155	11.5
General wards	902	36.8	440	32.7
**Paediatric**				
Emergency centre	71	2.9	47	3.5
ICU and HCU	221	9.0	97	7.2
General wards	144	5.9	70	5.2
**Neonatal general ward**	173	7.1	82	6.1
**Unknown ward location**	69	2.8	46	3.4
**Outpatients**	135	5.5	99	7.4

ICU, intensive care unit; HCU, high-care unit.

During the study period, 565 samples (23.1%) had a registered clinician. However, only 256 (10.4%) of the samples had an automatic SMS sent, and the remainder (*n* = 2195) were communicated telephonically. There was no significant difference between SMS communication compared to a telephone call with regard to the time taken to acknowledge on TrakCare^®^ (*p* = 0.454).

### Folder review results

One hundred and twenty (120) electronic patient records were evaluated. There was an equal distribution between the sexes: 59 (49.2%) women and 61 (50.8%) men. Two-thirds of patients (*n* = 80) were above 18 years of age. In 68.3% of the patients, no new management occurred after critical results were communicated by the laboratory (Table 3). Forty (33.3%) patients demised, where 31 (77.5%) were above the age of 50 years and 7 (17.5%) were under the age of 1 year. Derangements in potassium were found in half of the patients who demised; 14 patients (35.0%) had critical hyperkalaemia and 6 (15.0%) a critical hypokalaemia. Twelve patients (30%) had sodium derangements, where 9 patients (22.5%) had a critical hypernatraemia and 3 patients (7.5%) had a critical hyponatraemia. Of note, during the folder audit, 65 (54.2%) clinical decisions were based on point-of-care testing results. Point-of-care devices utilised included blood gas analysers (*n* = 52, 80%), glucometers (*n* = 5, 7.69%), and total cutaneous bilirubin meters (*n* = 8, 12.3%) (Table 3).

**TABLE 3:** Randomised folder audit of patients with critical results at Tygerberg Academic Hospital, South Africa, October 2021 to March 2022.

Variable	*n*	%
**Point-of-care device**	65	54.2
Blood gas	52	80.0
Glucometer	5	7.7
Bilirubinometer	8	12.3
**Management**		
Management initiated	38	31.7
No change in management	82	68.3
**Patient outcome**		
Discharged	69	57.5
Deceased	40	33.3
Transferred	10	8.3
Refused further care	1	0.8

## Discussion

In this study of time to clinician acknowledgement of critical laboratory results, we found that only two-thirds of these results were acknowledged within 24 h. Thus, the efficacy of telephonic communication, the method by which critical laboratory results are typically communicated at TAH, is unclear. Despite TrakCare^®^ sending an automatic SMS for critical results, we found there was no significant difference in the time to acknowledge these results compared to telephonic communication. Further investigation into the reasons for clinicians not being registered may improve this method of communication in our setting. Directly informing the primary treating on-call clinician of a critical result may be a more effective method; however, this may be challenging if the requesting clinician is not on duty and unavailable to accept the result.^7^ This is particularly challenging in the outpatient setting, where important results can only be communicated during business hours. Further engagement with these stakeholders may offer additional insight into improving communication of these results.

The time for clinician acknowledgement within 24 h ranged between 80 and 198 min across the different wards. A study in Boston, United States, reviewed 37 503 critical results reported from their core laboratory over a year and found that the average time to convey a critical result to the clinician or patient location was 22 min.^12^ This study found that factors associated with delayed reporting times included testing performed in outpatient departments (where staff are only available during office hours), as well as incomplete test request forms that lack the patient location or treating clinician details.^12^

A previous study at TAH laboratory assessing incomplete laboratory request forms and their impact on critical results had similar findings to our study.^13^ In this study, 2550 paper-based request forms were analysed; 61.2% had no clinician contact details, 4.9% had incomplete ward information, and a critical result was not communicated in 19.9% of cases.^13^ The present study found that 2.8% of critical results had no ward allocation, with these results having the longest inpatient laboratory turn-around time to communication. However, it did not have the longest time to clinician acknowledgement compared to the other wards. This may be attributed to the laboratory staff contacting the hospital’s central directory to determine the patient’s location and the on-call clinician’s contact details, which allows direct communication with the clinician.

At TAH, critical results are often not relayed directly to the treating clinician. A previous audit of critical result reporting at TAH found that treating clinicians received 12.7% of critical results and the remainder were given to ward staff such as nurses or clerks.^14^ A study by Howantiz et al. found that only 12.2% of critical results were received by clinicians, and they further reported that only 20.8% of nursing supervisors thought critical result lists were helpful, compared to 94.9% of clinicians.^15^

This gap in the knowledge of the impact of critical results may contribute to the delay in clinicians acknowledging these results. It also serves as a possible avenue for educational intervention in this regard. A retrospective study by Kuperman et al. reviewed the time for inpatients to be treated for critical results and found that the median time to intervention was 2.5 h.^16^ They reported that an important reason for the delay in intervention was that the primary clinician did not receive the information promptly.^16^

In our study, we found that only 23.1% of samples had a registered clinician and our laboratory often relies on the ward to communicate a result. Tygerberg Academic Hospital laboratory does not have direct access to the clinician on call and obtaining this information from the hospital’s central directory is time consuming. Schapkaitz et al. carried out a survey investigating critical value policies for haematology tests in private intensive care units in South Africa; 42.9% preferred the requesting clinician be contacted with the result and 28.6% stated that the on-call doctor should be contacted.^17^ Directly informing the primary treating or on-call clinician of a critical result may be a more effective method; however, this may be challenging if the requesting clinician is not on duty and is unavailable to accept the result.^7^

Alarmingly, a third of the patients in our folder audit demised. Thirty-five per cent had a critical hyperkalaemia result. A similar finding was reported by Kuo et al. in 2021 in a study conducted in Taiwan, where critically high potassium results were reviewed and a mortality of 34% was found.^18^ Additionally, we found that 30% of patients who demised had a critical sodium result, with hypernatraemia being more prominent (22.5%). A study in the United States in 2007 by Howanitz et al. evaluated critical sodium values in serum and whole blood over 6 months and found a mortality rate of 48% in those with critical hypernatraemia and 19% in those with critical hyponatraemia.^19^ The high mortality reported and reflected in these studies demonstrates the link between critical results and poor patient outcomes. This highlights the importance of regular auditing and improvement of critical results reporting from the laboratory. Clinician engagement and education may play a significant role in improving outcomes by ensuring that timeous management is initiated based on critical results.

Despite the high mortality in our study, we found that communication of critical laboratory results did not alter the management in over two-thirds of patients. This may be due to clinicians initiating empiric management while awaiting laboratory results, as we found the average time from samples being registered to being called from our laboratory ranged from 71 to 363 min. Of note, 54.2% of patients had a confirmatory point-of-care test performed where the clinician had already instituted management for the affected critical result. These devices are helpful in the clinical setting as they provide rapid availability of results. However, they carry limitations which clinicians may not be aware of. These include insufficient training of personnel utilising these instruments, a lack of quality control testing, and failure to partake in external quality control programmes.^20,21^ In our setting, point-of-care testing devices do not fall under the management of the central laboratory and results do not transmit to the LIS, thus the results of these tests were not added to our audit. Although the International Federation of Clinical Chemistry and Laboratory Medicine has established quality indicators for critical result reporting,^22^ our laboratory does not have these indicators in place.

### Recommendations

A survey will be sent to clinicians in the hospital to gain insight into the current practice of critical result reporting and the results will be used to develop a standard operating procedure and quality indicators. Additionally, a presentation on critical results will be provided for hospital staff, which will aim to educate them on their importance. After these interventions, a re-audit will be performed to determine their impact.

### Limitations

Patient data, collected from reviewing electronic patient records, may not have been accurately completed during the patient admission. The assumption was made that laboratory request forms, which were not manually checked during this study, were correctly captured onto the LIS. The patient’s location was based on the data captured on the LIS and may not reflect patient relocation to a different level of care before the critical result was available. Although different information systems are available for result notification, South Africa is a developing country with limited access to this type of infrastructure in the public healthcare setting. The lack of formal documentation for the time auxiliary staff communicated critical laboratory results to clinicians made it challenging to accurately reflect the clinician’s response time once alerted. Point-of-care testing is not under the management of our central laboratory and the results of these tests are not communicated to the LIS and have not been included in this audit. Lastly, downtime of TrakCare^®^ may have contributed to a delay in the time clinicians took to review results.

### Conclusion

To our knowledge, this is one of the few studies from Africa reviewing the use of critical values and the impact on patient care. Our study demonstrates the significance of timeous critical review verification on patient management. We will utilise our study findings to develop quality indicators for critical result reporting. The high mortality we reported, which is echoed in other studies, shows the need for collaboration between the laboratory and clinicians, and highlights the importance of establishing and maintaining this relationship to improve patient outcomes.
